# A Straight Skeleton Based Connectivity Restoration Strategy in the Presence of Obstacles for WSNs

**DOI:** 10.3390/s17102299

**Published:** 2017-10-10

**Authors:** Xiaoding Wang, Li Xu, Shuming Zhou

**Affiliations:** 1 School of Mathematics and Computer Science, Fujian Normal University, Fuzhou 350007, China; wangdin1982@fjnu.edu.cn (X.W.); zhoushuming@fjnu.edu.cn (S.Z.); 2 School of Online and Continuing Education, Fujian University of Technology, Fuzhou 350118, China

**Keywords:** connectivity restoration, representatives, obstacles, straight skeleton, WSNs

## Abstract

Connectivity has significance in both of data collection and aggregation for Wireless Sensor Networks (WSNs). Once the connectivity is lost, relay nodes are deployed to build a Steiner Minimal Tree (SMT) such that the inter-component connection is reestablished. In recent years, there has been a growing interest in connectivity restoration problems. In previous works, the deployment area of a WSN is assumed to be flat without obstacles. However, such an assumption is not realistic. In addition, most of the existing strategies chose the representative of each component, which serves as the starting point of relay node deployment during the connectivity restoration, either in a random way or in the shortest-distance based manner. In fact, both ways of representative selection could potentially increase the length of the SMT such that more relay nodes are required. In this paper, a novel connectivity restoration strategy is proposed—Obstacle–Avoid connectivity restoration strategy based on Straight Skeletons (OASS), which employs both the polygon based representative selection with the presence of obstacles and the straight skeleton based SMT establishment. The OASS is proved to be a 3-opt approximation algorithm with the complexity of O(nlogn), and the approximation ratio can reduce to $\frac{3\sqrt{3}}{2}$ while it satisfies a certain condition. The theoretical analysis and simulations show that the performance of the OASS is better than other strategies in terms of the relay count and the quality of the established topology (i.e., distances between components, delivery latency and balanced traffic load) as well.

## 1. Introduction

Wireless sensor networks (WSNs) are well-known by their significant advantages in monitoring, such as battlefield surveillance, environment monitoring and biological detection. To achieve events surveillance, a large amount of sensors are intentionally placed to monitor a certain geographic area. All data collected are forwarded toward a base station along at least a multi-hop wireless path. In this case, the reachability between any pair of sensors implies the importance of the network connectivity. Due to such special architecture, WSNs are more vulnerable to human sabotages and natural disasters that can partition a WSN into disjointed components. Once the connectivity is lost, the data will be no longer sent back to the center that will cause severe consequences.

Due to the significance of network connectivity as mentioned above, the problem of connectivity restoration has been receiving increasing attention in recent years. All known solutions consider how to reestablish the connectivity through building a Steiner Minimal Tree (SMT) among all components with the minimum cost, which includes relay device consumption and energy consumption [1]. According to the classification of the cost, there exist two major types of restoration strategies. One utilizes the least number of relay devices (i.e., Relay Nodes (RNs) and Mobile Data Collectors (MDCs)) to federate disjointed components [2,3,4,5,6,7,8,9,10,11,12,13,14,15,16,17,18]. However, most strategies of this type choose the representative either in a random way or in the shortest-distance based manner. In fact, both ways of representative selection could potentially increase the length of the SMT such that more RNs are required. In addition, they assume the deployment area of a WSN is flat without obstacles. Note that, if there are no obstacles between two nodes, then a signal originated from one node could be received by the other one even with a slight signal degradation caused by a long distance transmission. On the other hand, another type of connectivity restoration strategy pursues the minimum energy consumption influenced by a variety of realistic terrains (e.g., mountain, river, forest, swamp, etc.) [19,20,21,22,23]. In this paper, the connectivity restoration strategy with minimum RN consumption is developed. In addition, we consider the mountain as the only available terrain factor. If there exist buildings and other structures along with mountains that are big enough and hard to penetrate, then all of these objects are considered as impenetrable obstacles.

### Our Contribution

This paper presents an Obstacle–Avoid connectivity restoration strategy based on Straight Skeletons, namely OASS, which consists of an adapted minimum spanning tree construction algorithm Ad-Prim, an obstacle–avoid algorithm OA and a straight skeleton based SMT construction algorithm SSIN. The details of our contributions are listed as follows:

1. We modify the well-known Minimum Spanning Tree (MST) construction algorithm Prim to a circle-tolerant one, namely Ad-Prim, by choosing multiple nodes on the perimeter of each component as corresponding representatives such that the MST is constructed with the rule that circles formed by nodes on the same components should be neglected. Compared with other strategies that choose only one node for each component as its representative, the spanning tree established by Ad-Prim is much shorter.
2. We develop an obstacle–avoid algorithm, OA, to deal with the case that there exist obstacles lying on the line-segment between each pair of nodes. Instead of choosing a detour going along the perimeter of an obstacle, the candidate point on the perimeter of the obstacle is carefully chosen such that a shortest path around the obstacle consists of at least two line-segments tangent at such point. In addition, compared with other strategies disregarding the presence of obstacles, OA can be applied to more realistic scenarios.
3. We devise a straight skeleton based algorithm, SSIN, to build an SMT such that the shortest inter-component connection is achieved. Each straight skeleton will be placed within the potential convex hull of MST as the deployment route for RNs. Furthermore, if multiple line-segments between pairs of nodes have obstacles, then the SSIN is an option to avoid obstacles.
4. We prove that OASS is a 3-opt approximation algorithm with the complexity of O(nlogn), and the approximation ratio can reduce to $\frac{3\sqrt{3}}{2}$ while the network topology can be decomposed into a series of convex hulls. The theoretical analysis and simulations show that the performance of OASS is better than other strategies, especially a series of which deploy RNs toward the center of the deployment area, not only in the number of RNs required but the quality of the established topology (i.e., distances between components, delivery latency and balanced traffic load) as well.

The rest of the paper is organized as follows. Related work is covered in Section 2. The notions, terminologies and the problem description are introduced in Section 3. The strategy is elaborated in Section 4. Section 5 gives the theoretical analysis on approximation radio and complexity of OASS. In addition, the validation results are presented in this section as well. We conclude this paper in Section 6.

## 2. Related Works

According to the classification of the cost, there exist two major types of restoration strategies [1]. One utilizes the least number of relay devices (i.e., RNs and MDCs) to federate disconnected components [2,3,4,5,6,7,8,9,10,11,12,13,14,15,16,17,18], while the other one pursues the minimum energy consumption influenced by realistic terrains [19,20,21,22,23].

Due to the variety of relay devices, strategies based on relay device consumption can be subdivided into two categories. One aims to deploy the minimum number of RNs to reestablish connectivity as follows, the performance of which are always measured by the approximation ratio [2,3,4,5,6,7,8,9,10]. In [2], Chen et al. first prove the MST based approach is 3-opt and then they propose a 4-star based 3-opt algorithm. Similarly, Cheng et al. [5] employ 3-star to connect disjointed components and then design a 3-hypergraph based algorithm, the approximation ratios of which are 3 and 2.5, respectively. It is worth mentioning that, although 2.5 is the minimum one among all approximation ratios, the complexity of the 3-hypergraph based algorithm is $O(n^{3}q_{p}^{2})$, where $q_{p}=\max\left\{q_{a,b,c}|a,b,c\subset P\right\}$, $q_{a,b,c}$ denotes the number of RNs required to connect nodes *a*, *b*, *c* and *P* represents the set of disconnected nodes. In [4], Lloyd et al. give an improved MST based algorithm with a approximation ratio 7. Tang et al. [3] map the deployment area into a series of regular cells, by placing a cluster head node within each cell in charge with inter-cell communications the connectivity is restored and the approximation ratio of which is 4.5. Efrat et al. [10], Yang et al. [7] and Misra et al. [6,8] give weight values to different types of connections (i.e., node to node, node to relay and relay to relay) to build a weighted complete graph. Then, they employ the construction algorithm of the minimum weight Steiner tree [24] to restore the connectivity in different scenarios, the approximation ratio of this algorithm for each scenario is 3.11, 6.43, 6.2 and 12.4, respectively, and the complexities for all scenarios are at least ${O(|S|}^{k}{.|V-S|}^{k-2}+{k.|S|}^{2k+1}\log|S|)<O\left(n^{2k-2}+n^{2k+1}\log n\right)$, where S⊂V denotes the set of nodes required to be connected, |V|=n and k≥3. Recently, Wang et al. [9] proposes a 3-opt algorithm based on the center of mass and 3-star. There are some efficient algorithms without giving approximation ratios [11,12,13,14,15,16,17,18]. Lee et al. propose efficient algorithms in [13,15], respectively, to deploy RNs toward the center of the deployment area. In [14], Ranga et al. first construct a distance function, then based on the zero gradient point of which the relay node deployment route is planned. Joshi et al. [12] employ the straight skeleton as the relay node deployment route. In [11], Senel et al. design two efficient algorithms. One is an optimal triangle construction algorithm based on the MST, while the other one is based on Delaunay triangulation, both of which employ Fermat points to achieve the connectivity restoration. In [16], Uwitonze et al. employ space network coding, Delaunay triangulation, non-uniform partitioning techniques and linear programming to design a relay node deployment route. On the other hand, another type of strategy based on relay devices’ consumption considers the situation that MDCs serve as a channel to offer intermitted connection between disjoint components while only insufficient RNs are available [17,18]. However, these types of strategies always evaluate the performances through simulations. Both Joshi et al. [18] and Abbas et al. [17] partition the set of components into a number of convex hulls and build an MST for inter-partition connection. Then, they employ convex hulls as routes for MDCs, which are shortened in [18] through an angle bisector based optimization approach, in order to provide intermitted connections. At last, the connectivity is restored by deploying RNs along the MST. It is worth mentioning that OASS solves both of the representative selection problem and the obstacle–avoid problem; although neither of them are taken into consideration by all of these works above. Furthermore, although the optimal approximation ratio of OASS is $\frac{3\sqrt{3}}{2}$, which is a little higher than 2.5, the complexity of OASS is only O(nlogn). The comparison of some contemporary heuristic algorithms for connectivity restoration in WSNs through RNs placement is shown in Table 1.

There are many terrain based connectivity restoration strategies. In [20], Senturk et al. quantifies the influence of realistic terrains through a grid based mapping. Then, they design the ReBAT that considers realistic terrains such that the least cost paths for connectivity restoration is built. Similarly, Wang et al. [23] devise a hybrid strategy to achieve the 2-connectivity restoration based on realistic terrains. Zhou et al. [19] propose an extended Rapidly exploring Random Tree (RRT) based algorithm to find a least cost path to avoid obstacles and federate components. Truong et al. [21] design a family of algorithms that consider the impact of obstacles on mobility and communication to restore the connectivity with a minimum number of RNs and meanwhile minimizes the mobility cost of agents. In [22], Mi et al. investigate how to avoid convex obstacles and inter-sensor collisions during connectivity restoration. Unlike these works, only one terrain factor, the mountain, is considered as an impenetrable obstacle by OASS.

## 3. Preliminary

### 3.1. System Model

The disconnected network is mapped to a graph G(V,E). Each node $s_{i}\in V$ represents a sensor with a communication rage *r*, while $s_{i}s_{j}\in E$ denotes the communication link between a pair of sensors $s_{i}$ and $s_{j}$. Since the network is partitioned into a number of disjoint components $C_{i}$s, all $s_{i}s\in C_{i}$ remain connected. Some notations used throughout this paper are given first, and the important symbols with their definitions are collected in Table 2.

**Definition** **1.** [25] A Euler Closed Trail, abbreviated as ect, is a closed trail that visits every edge of graph G exactly once.

**Definition** **2.** A potential Convex Hull, abbreviated as pch, is a path $s_{1}s_{2}\ldots s_{k}$ such that $s_{1}s_{k}{⋃}_{i=1}^{k-1}s_{i}s_{i+1}$ is a convex hull of the set of nodes $\left\{s_{i}|1\leq i\leq k\right\}$.

**Definition** **3.** [26] The straight skeleton of a polygon is defined by a continuous shrinking process in which the edges of the polygon are moved inwards parallel to themselves at a constant speed. As the edges move in this way, the vertices where pairs of edges meet also move, at speeds that depend on the angle of the vertex. If one of these moving vertices collides with a nonadjacent edge, the polygon is split in two by the collision, and the process continues in each part. The straight skeleton is the set of curves traced out by the moving vertices in this process.

The shortest SMT considered as the shortest inter-component connection has the following property [27]. Note that each terminal denotes to a representative of a $C_{i}$, while each Steiner point represents a rendezvous point at which three edges are adjacent to each other.

**Property** **1.**

*1.*  Each terminal is a leave and each Steiner point connects to exactly three terminals.
*2.*  The angle between each pair of adjacent edges at a Steiner point is exactly $120^{\circ }$.
*3.*  There are totally n−2 Steiner points for n terminals.

### 3.2. Problem Statement

This paper is dedicated to the connectivity restoration problem, which is formally stated as follows:

Given a graph *G* with *n* disjoint components $C_{i}$s that consist of sensors with a transmission range *r* and obstacles *O*s, the goal is to provide an efficient solution that ensures that *n* components will be 1-connected by deploying RNs at an interval *r* as few as possible. That is,

(1) $$\begin{array}{cc} min\; & N_{A}\\ st.\; & 1.\;\exists Os,\\ & 2.\;\forall s_{i}\in C_{i},s_{j}\in C_{j},\\ & \;\;\;\;\exists P_{s_{i},s_{j}}\neq ⌀, \end{array}$$

where $N_{A}$ denotes the number of RNs required by employing approach *A*.

## 4. The OASS Approach

As a three-phase strategy, OASS is devised to cope with the connectivity restoration problem with the presence of obstacles. In phase one, an adapted minimum spanning tree construction algorithm Ad-Prim is designed to deal with the improper representative selection problem. In phase two, an obstacle–avoid algorithm OA is developed to build the shortest paths around obstacles. In the last phase, a straight skeleton based algorithm SSIN is conceived to achieve the shortest inter-component connection and obstacles avoidance.

### 4.1. Adapted Minimum Spanning Tree Construction Algorithm (Ad-Prim)

Before we employ the straight skeleton based RNs deployment to achieve the establishment of inter-component connection, the candidate positions for the placement of straight skeletons, which are potential convex hulls, should be properly chosen along the MST of all disjointed components. The reason for finding the shortest MST is that, if a longer MST is built, then the area of each potential convex hull could be enlarged such that the length of each corresponding straight skeleton is increased. No double that a longer MST will eventually result in more RNs required. In fact, although the MST construction algorithm Prim [24] helps to locate the tree with minimum length, without properly chosen representatives, the generated MST is not the shortest one. Therefore, the selection for eligible representatives are crucial during the MST construction.

In previous works, there exist two methods of representative selection. The first one [2,4,5,6,7,8,10,11,12,17,18] is randomly choosing a node within a component to represent it, while the second one [9,13,15] chooses a number of nodes, one from each component, closest to each other as corresponding representatives. If the Prim is used to find an MST, then it is obvious that the latter one will result in a shorter MST. However, both methods can hardly build the shortest MST, since choosing multiple nodes as representatives of a $C_{i}$ can potentially shorten inter-component distances. Due to the reasons explained above, we adapt the Prim to a circle-tolerant algorithm, namely Ad-Prim. The Ad-Prim will generate the shortest MST through the following steps.

1. The perimeter of each component $C_{i}$ is modelled to a polygon $P_{C_{i}}$. All nodes on the $P_{i}$ are the representatives of $C_{i}$.

2. All representatives and the shortest paths between them around obstacles found by OA are prepared for the MST construction in the next step.

3. The Prim is employed to build an MST among all representatives with one rule that each circle formed by nodes on the same component should be neglected.

According to the description above, the pseudo code of Ad-Prim is given as Algorithm 1.

**Algorithm 1** Adapted Minimum Spanning Tree Construction Algorithm Ad-Prim.**Input:** all nodes on the perimeter of all components and a set of shortest paths $\left\{P_{s_{i}s_{j}}|1\leq i,j\leq n\right\}$**Output:** an MST   choose all nodes on $P_{C_{i}}$ as representatives of $C_{i}$   employ Prim to construct an MST among all representatives with one rule that each circle formed by nodes on the same component should be neglected   **return** an MST

Although the proper selection of multiple representatives for each component helps to build the shortest MST, there is a chance that an edge $s_{i}s_{j}\in MST$ may intersect with an obstacle. This requires an obstacle–avoid algorithm.

### 4.2. Obstacle–Avoid Algorithm (OA)

**Figure 1:**
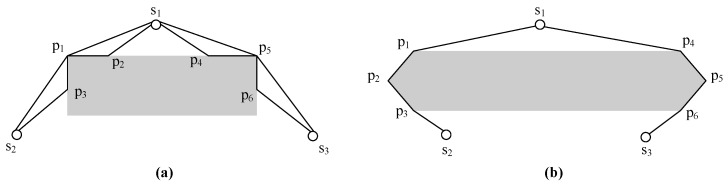
The shortest paths around the obstacle w.r.t (**a**) when there is only one point; (**b**) when there are more points sitting in the middle.

In a real scenario, there are always some obstacles on the deployment area. If an obstacle lies right on the middle of two disjointed nodes, then the line-segment between such pair of nodes for RNs deployment is not an option. In this case, although a detour going along the perimeter of the obstacle could be a solution, yet it is not the best choice. In fact, the shortest detour between a pair of nodes with an obstacle sitting right on the middle consists of a series of paths with at least two tangents involved. To be more specific, an obstacle is modelled to a polygon first. Then, the convex hull based on the polygon of the obstacle is constructed. At last, by carefully choosing the line-segments between two nodes and the points on the convex hull, the shortest path around the obstacle will be found. Note that there are two cases that should be considered. One is the shortest path composed of only two tangents between a specific point on the convex hull and two nodes, respectively (see Figure 1a). The other one not only includes all paths in the previous case but involves more paths between multiple points on the convex hull (see Figure 1b) as well. Furthermore, since an MST intersecting with obstacles is built, it is much easier to deal with each edge $s_{i}s_{j}\in MST$ intersecting with an obstacle in order to construct the $P_{s_{i},s_{j}}^{O}$.

The OA will find shortest paths through the following steps.

1. For two nodes $s_{1}$, $s_{2}$ and an obstacle *O*, find two points *x*, *y* on $CH_{O}$, such that there exist two tangents $s_{1}x$ and $s_{2}y$.

2. If x=y, then $s_{1}xs_{2}$ is the $P_{s_{1}s_{2}}^{O}$. Otherwise, let the set of points $S=\{x_{i}|0\leq i\leq k-1\}$ denote the least number of points from *x* to *y* on $CH_{O}$ where $x_{0}=x$ and $x_{k}=y$. Then, $P_{s_{1}s_{2}}^{O}=s_{1}x⋃ys_{2}{⋃}_{i=0}^{k-1}x_{i}x_{i+1}$.

According to the description above, the pseudo code of OA is given as Algorithm 2.

**Algorithm 2** Obstacle–Avoid Algorithm OA.**Input:** the set of edges $\left\{s_{i}s_{j}|i\neq j,1\leq i,j\leq n\right\}$**Output:** each a set of shortest paths around obstacles $\left\{P_{s_{i}s_{j}}^{O}|1\leq i,j\leq n\right\}$  **for** each pair of nodes $s_{i}$ and $s_{j}$
**do**    **if** there is an obstacle *O* on the line-segment $s_{i}s_{j}$
**then**        find two points *x* and *y* on $CH_{O}$ such that there exist two tangents $s_{1}x$ and $s_{2}y$        using the least number of points $\left\{x_{i}|x_{0}=x,x_{k}=y,0\leq i\leq k-1\right\}$ on $CH_{O}$ to construct $P_{s_{1}s_{2}}^{O}=s_{1}x⋃ys_{2}{⋃}_{i=0}^{k}x_{i}x_{i+1}$    **end if**  **end for**  **return** a set of shortest paths around obstacles $\left\{P_{s_{i}s_{j}}^{O}|1\leq i,j\leq n\right\}$

### 4.3. Straight Skeleton Based SMT Construction Algorithm (SSIC)

**Figure 2:**
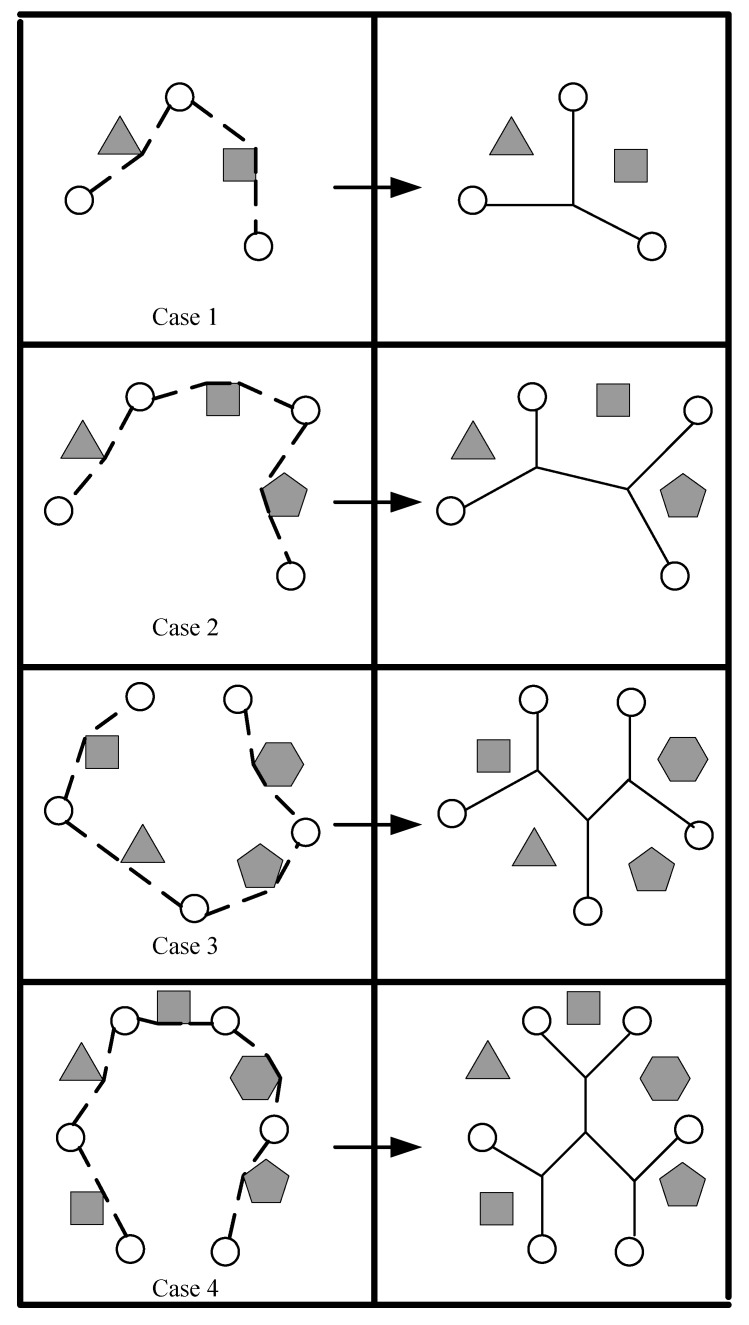
The obstacle avoidance by straight skeletons.

Although deploying RNs along the MST can accomplish the connectivity restoration, yet it consumes a large number of RNs. In order to reduce the consumption of RNs, a better way is required that can offer a connection structure simpler and shorter than the MST. In fact, a straight skeleton is a tree such that all terminals are leaves and each pair of adjacent edges at a Steiner node has an angle of exactly $120^{\circ }$ [28]. Such characteristics satisfy property 1. Therefore, the straight skeleton can reduce the length of the inter-component connection, which implies the RNs required are less than that of the MST. Before we apply the straight skeleton to connectivity restoration, we need to know where a straight skeleton should be placed. More importantly, using a convex hull to generate a straight skeleton only costs O(nlogn) [28]. There is no doubt that each convex hull should be found to place a straight skeleton. In fact, when the MST is built, every convex hull will be located along the MST. Furthermore, if there exist obstacles between pairs of nodes, then the straight skeleton is an option to avoid obstacles (see Figure 2).

Next, we will show how SSIC achieves the construction of the straight skeleton based inter-component connection through the following steps.

1. A leave node $s_{1}$ on the MST is randomly chosen as the starting node to draw a Euler closed trail $ect=s_{1}s_{2}\ldots s_{n}s_{1}$ of the MST, where *n* denotes the number of representatives.

2. Starting from $s_{1}$, we try to locate every potential convex hull $pch_{i}$ along ect. If there exists an edge $s_{i}s_{i+1}\in pch_{i}$ such that $s_{i}s_{i+1}=s_{i}p_{i}⋃p_{i}p_{i+1}\ldots ⋃p_{i+k-1}p_{i+k}⋃p_{i+k}s_{i+1}$, then let $pch_{i}=⋃p_{i}p_{i+1}⋃pch_{i}$. It is worth mentioning that, if there is a $pch_{i}=s_{i}s_{i+1}\cdots s_{i+k}$ such that the set $S=\{s_{i+j}|1\leq j\leq k\}$ of nodes are on the $C_{i}$, then let $pch_{i}=s_{i}s_{i+1}\ldots s_{i+j}$ and choose $s_{i+k}$ to proceed. Otherwise, we start from $s_{i+k+1}$ and repeat the pch localization process until all representatives are looped over.

3. For each $pch_{i}$, if there are no obstacles intersecting with the straight skeleton within the $pch_{i}$, then this straight skeleton serves as the deployment route for RNs. Otherwise, according to the number *k* of obstacles, we split the $pch_{i}$ into k+1 parts such that $pch_{i}={⋃}_{i=1}^{k+1}pch_{i}^{j}$ and there exists at least one part, say $pch_{i}^{j}$, the straight skeleton of which is not intersecting with obstacles. Then, we place RNs along the straight skeleton inside the $pch_{i}^{j}$. If there exists a *k* such that k≠j, then each edge $e\in pch_{i}^{k}$ is a relay node deployment route. It is worth mentioning that all straight skeletons can be built at $pch_{i}$s once and for all.

According to the description above, the pseudo code of SSIN is given as Algorithm 3.

**Algorithm 3** Straight Skeleton based SMT Construction Algorithm (SSIC).**Input:** an MST that consists of *n* representatives**Output:** a straight skeleton based tree   randomly choose a leave node $s_{1}\in MST$ to draw an euler closed trail $ect=s_{1}s_{2}\ldots s_{n}s_{1}$   **for** all $s_{i}\in ect$
**do**        **if** there exists an edge $s_{i}s_{i+1}\in pch_{i}$ such that $s_{i}s_{i+1}=s_{i}p_{i}⋃p_{i}p_{i+1}\ldots ⋃p_{i+k-1}p_{i+k}⋃p_{i+k}s_{i+1}$
   **then**
$pch_{i}=⋃p_{i}p_{i+1}⋃pch_{i}$        **end if**        **if** there is a $pch_{i}=s_{i}s_{i+1}\cdots s_{i+k}$ such that $S=\{s_{i+j}|s_{i+i}\in C_{i},1\leq j\leq k\}$
**then**
$pch_{i}=s_{i}s_{i+1}\ldots s_{i+j}$ and $s_{i}=s_{i+k}$        **else**$\;\;s_{i}=s_{i+k+1}$        **end if**   **end for**   **for** all $pch_{i}$s **do**        **if** there is no obstacles intersecting with the straight skeleton within the $pch_{i}$
**then**          the straight skeleton of the $pch_{i}$ is a relay node deployment route        **else** split the $pch_{i}$ into k+1 parts such that $pch_{i}={⋃}_{i=1}^{k+1}pch_{i}^{j}$             **if** there exists a $pch_{i}^{j}$, the straight skeleton of which is not intersecting with obstacles **then**               the straight skeleton will be the route for placing RNs             **end if**             **for** other $pch_{i}^{k}$s, k≠j, **do** each edge $e\in pch_{i}^{k}$ is a relay node deployment route             **end for**        **end if**   **end for**   **return** a straight skeleton based tree

**Figure 3:**
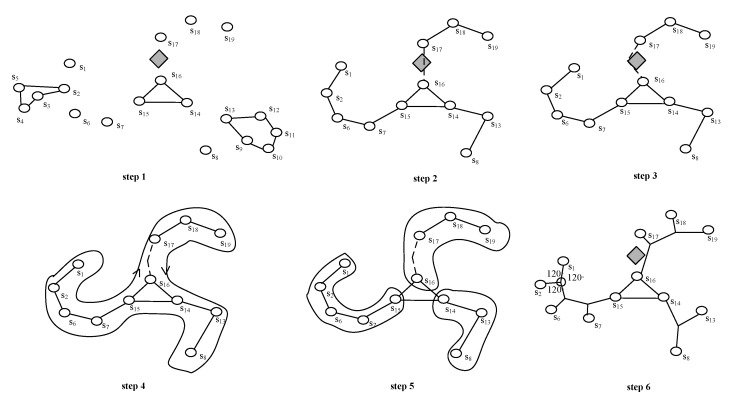
An example of Obstacle–Avoid connectivity restoration strategy based on Straight Skeletons (OASS).

Next, we give an example of how to restore the connectivity of a disconnected network. As shown in step 1 of Figure 3, there are 19 sensor nodes that consist of 10 components. Since these 19 sensor nodes are on the perimeter of all 10 components, all sensor nodes serve as representatives. Obviously, there is an obstacle on the line-segment between $s_{16}$ and $s_{17}$. In step 2 of Figure 3, the MST of 12 representatives are built using Ad-prim with three circles $s_{14}s_{15}s_{16}s_{14}$, $s_{2}s_{3}s_{4}s_{5}s_{2}$ and $s_{13}s_{9}s_{10}s_{11}s_{12}s_{13}$ neglected. Note that sensor nodes $s_{3}$, $s_{4}$, $s_{5}$, $s_{9}$, $s_{10}$, $s_{11}$ and $s_{12}$ are no longer useful in the following process; therefore, we ignore them for simplicity. As shown in step 3 of Figure 3, since there is only one path $s_{16}s_{17}$ intersecting with the obstacle, we use OA to locate the $P_{s_{16}s_{17}}^{O}$ which consists of two tangents $s_{16}x$ and $xs_{17}$. In step 4 of Figure 3, SSIC first randomly chooses a leave node, say $s_{1}$, as the beginning to draw the euler closed trail $ect=s_{1}s_{2}s_{6}s_{7}s_{15}s_{16}s_{17}s_{18}s_{19}s_{18}s_{17}s_{16}s_{14}s_{13}s_{8}s_{13}s_{14}s_{15}s_{7}s_{6}s_{2}s_{1}$. As shown in step 5 of Figure 3, three potential convex hulls $s_{1}s_{2}s_{6}s_{7}s_{15}$, $s_{19}s_{18}s_{17}s_{16}$ and $s_{14}s_{13}s_{8}$ are found along the ect. Note that, although $s_{1}s_{2}s_{6}s_{7}s_{15}s_{16}s_{1}$, $s_{19}s_{18}s_{17}s_{16}s_{14}s_{19}$ and $s_{14}s_{13}s_{8}s_{15}s_{14}$ can form tree convex hulls respectively, edges $s_{15}s_{16}$, $s_{16}s_{14}$ and $s_{14}s_{15}$ belong to a component, for which such three edges should be removed form three pchs. At last three straight skeletons are placed within three pchs since there are no obstacles intersecting with straight skeletons as shown in step 6 of Figure 3. Eventually, RNs are deployed along the straight skeleton based tree to restore the connectivity.

## 5. Performance Analysis

### 5.1. Theoretical Analysis

In this section, we give the theoretical analysis on OASS about the approximation ratio, the advantage over a series of connectivity restoration strategies in terms of RNs required and the complexity.

We call all approaches $A_{rc}$, if they either randomly choose a node from each component as the corresponding representative or select a number of nodes, each from a component, closest to each other as representatives [2,4,5,6,7,8,9,10,11,12,13,15,17,18]. Let $L_{MST_{rc}}$ denote the length of an MST built by $A_{rc}$. For a graph *G*, we have the following theorem holding true.

**Theorem** **1.** *For a graph G, if $t_{}$ is a tree constructed by Ad-Prim, then we have following two results:*

*1.*  $t_{}$ is the MST of G.
*2.*  $L_{t_{}}\leq L_{MST_{rc}}$.

**Proof.**  First, we prove result 1 by contradiction.

**Figure 4:**
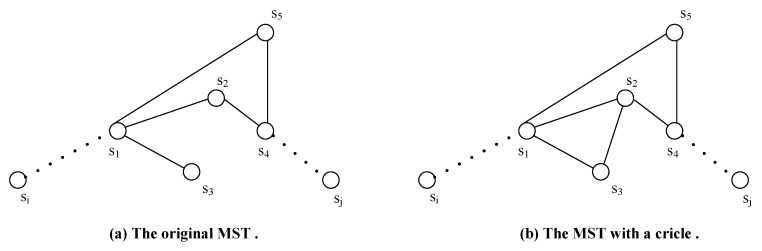
Adding a circle to a MST.

We are going to take Figure 4 as an example to complete the proof. Supposing that *t* is not the MST of graph *G* with $s_{1}s_{2},s_{1}s_{3}\in t$, let $t^{*}$ denote the MST of graph *G* with $s_{1}s_{2},s_{2}s_{3}\in t^{*}$. It is obvious that $t⋃t^{*}$ has at least a circle $s_{1}s_{2}s_{3}s_{1}$. Next, we try to distinguish three cases to prove the first result.

**Case 1**: $L_{s_{2}s_{3}}<L_{s_{1}s_{3}}$.

If $L_{s_{2}s_{3}}<L_{s_{1}s_{3}}$, then $s_{2}s_{3}$ should be chosen first, we can get a shorter $t=t\setminus s_{1}s_{3}⋃s_{2}s_{3}$, this contradicts the structure of *t*.

**Case 2**: $L_{s_{1}s_{3}}=L_{s_{2}s_{3}}$.

If $L_{s_{1}s_{3}}=L_{s_{2}s_{3}}$, then we have $t=t^{*}$.

**Case 3**: $L_{s_{2}s_{3}}>L_{s_{1}s_{3}}$.

If $L_{s_{2}s_{3}}>L_{s_{1}s_{3}}$, then we can get a shorter $t^{*}=t^{*}\setminus s_{2}s_{3}⋃s_{1}s_{3}$. This contradicts the assumption that $t^{*}$ is the MST of *G*.

Then, we prove result 2.

**Figure 5:**
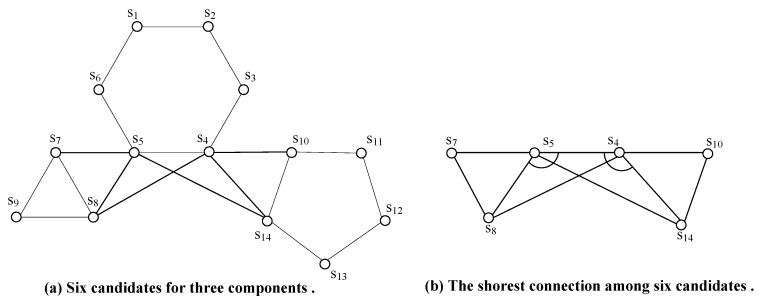
The representative selection.

We are going to take Figure 5 as an example to complete the proof. Let three components $C_{1}$, $C_{2}$ and $C_{3}$ consist of $s_{1}s_{2}\ldots s_{6}$, $s_{7}s_{8}s_{9}$ and $s_{10}s_{11}\ldots s_{14}$, respectively, and then the closest nodes be chosen as representative based on $A_{rc}$ are $s_{5}$, $s_{8}$ and $s_{14}$ for $C_{1}$, $C_{2}$ and $C_{3}$, respectively. The line-segment $s_{4}s_{5}$ extends to the left and right until it reaches the $s_{7}$ and $s_{10}$, respectively. By adjusting the positions of $C_{2}$ and $C_{3}$, we have $L_{s_{5}s_{7}}=L_{s_{5}s_{8}}$ and $L_{s_{4}s_{10}}=L_{s_{4}s_{14}}$ and both of $∠s_{8}s_{5}s_{4}$ and $∠s_{5}s_{4}s_{14}$ are obtuse. This implies that $L_{s5s_{14}}>L_{s_{4}s_{14}}=L_{s_{4}s_{10}}$. Since Ad-Prim could choose multiple nodes, say $s_{4}$ and $s_{5}$, as representatives of $C_{1}$, the tree *t* built by Ad-Prim could consist of $s_{5}s_{7}$ and $s_{4}s_{10}$. This implies $L_{t_{}}\leq L_{MST_{rc}}$.  ☐

We call all approaches $A_{tc}$, if they deploy RNs toward the center of either the deployment area or the polygon of all representatives to build a center-toward tree for connectivity restoration [9,13,14,15], while a series of MST based approaches [2,4,17,18] are called $A_{MST}$.

**Theorem** **2.** *For a graph G, relay node consumption function with respect to the number n of representatives of G and the length $L_{t}$ of a tree t constructed by $A_{MST}$, OASS and $A_{tc}$, respectively, are given as follows:*

1. $N_{A_{MST}}=\frac{L_{t}-2r\left(n-2\right)-2r}{r},$
2. $N_{OASS}=\frac{L_{t}-3r\left(n-2\right)-nr}{r}+n-2,$
3. $N_{A_{tc}}=\frac{L_{t}-2rn}{r}+1.$

**Proof.**  According to the variety of structures of trees established by different approaches, besides RNs being deployed along a planned route at an interval *r* to restore the connectivity, we can infer each unique relay node consumption function with respect to the number of representatives *n* and the length $L_{t}$ of a tree *t* constructed by OASS, $A_{tc}$ and $A_{MST}$, respectively. In addition, we consider the situation that the topology of a graph *G* can be decomposed to a number of convex hulls, which implies $MST=⋃pch_{i}$. Based on each subtree $pch_{i}\in MST$, we distinguish three cases to prove this theorem.

**Case 1**: Since each $pch_{i}$ is on the perimeter of a convex hull and RNs are placed on each edge $s_{i}s_{j}\in pch_{i}$ starting from $s_{i}$ and $s_{j}$ at the interval *r*, relay node consumption function $N_{A_{MST}}$ is given as follows:

(2) $$\begin{array}{c} N_{A_{MST}}=\frac{L_{t}-2r\left(n-2\right)-2r}{r}. \end{array}$$

**Case 2**: If there exist two potential convex hulls $pch_{i}$ and $pch_{j}$ such that $pch_{i}⋂pch_{j}=s_{k}$, then both of $pch_{i}$ and $pch_{j}$ remain the same. The tree *t* built by OASS consists of a number of straight skeletons. Since a straight skeleton has property 1 described in Section 3, each Steiner point requires an RN that implies totally n−2 RNs needed. In addition, these Steiner nodes serve as the starting point to deploy RNs toward all *n* leave nodes. Therefore, relay node consumption function $N_{}$ is given as follows:

(3) $$\begin{array}{c} N_{OASS}=\frac{L_{t}-3r\left(n-2\right)-nr}{r}+n-2. \end{array}$$

**Case 3**: For a center-toward tree *t*, if there exist two potential convex hulls $pch_{i},pch_{j}\in t$ such that $pch_{i}⋂pch_{j}=s_{k}$, then let $pch_{j}=pch_{j}\setminus s_{k}$. By doing so, all $pch_{i}$s can be separated from each other such that each leave node $s_{i}\in pch_{i}$ can deploy RNs toward the center. Since $A_{tc}$ always have a center, at least a RN is required at the position of the center as the beginning in order to deploy RNs towards all *n* leave nodes. Therefore, relay node consumption function $N_{A_{tc}}$ is given as follows:

(4) $$\begin{array}{c} N_{A_{tc}}=\frac{L_{t}-2rn}{r}+1. \end{array}$$

  ☐

Then, we prove the relationships between three relay node consumption functions $N_{OASS}$, $N_{A_{tc}}$ and $N_{A_{MST}}$.

**Theorem** **3.** *$N_{OASS}\leq N_{A_{tc}}\leq N_{A_{MST}}.$*

**Proof.**  For simplicity, we let r=1. According to Equations (2)–(4), we have the following results:

(5) $$\begin{array}{ccc} L_{t}-3n+4 & = & N_{OASS}, \end{array}$$

(6) $$\begin{array}{ccc} L_{t}-2n+2 & = & N_{A_{MST}}, \end{array}$$

(7) $$\begin{array}{ccc} L_{t}-2n+1 & = & N_{A_{tc}}. \end{array}$$

Since each pch consists of at least three nodes, we have n≥3. Therefore, the following inequations hold true:

(8) $$\begin{array}{c} \begin{array}{cc} N_{OASS} & =L_{t}-3n+4,\\ & <L_{t}-2n+1=N_{A_{tc}},\\ & <L_{t}-2n+2=N_{A_{MST}}. \end{array} \end{array}$$

In addition, for all *r*, r>1, it is easy to verify the correctness of this theorem.  ☐

**Theorem** **4.** [2] The approximation ratio of $A_{MST}$ is 3.

According to Theorem 3, it is easy to deduce that, if RNs are equally employed, then we have $L_{ss}<L_{tc}<L_{MST}$, where $L_{ss}$, $L_{tc}$ and $L_{MST}$ denote the tree constructed by OASS, $A_{tc}$ and $A_{MST},$ respectively.

**Theorem** **5.** The approximation ratio of OASS is 3.

**Proof.**  The OASS builds an obstacle–avoid MST first, then subdivides the MST into a number of subtrees. If a subtree t∈MST is a pch, then a straight skeleton is placed within as a relay node deployment route. Otherwise, RNs are deployed along *t*. We distinguish the following two cases to prove this theorem.

**Case 1**: the topology of graph *G* can be decomposed to a series of convex hulls.

In this case, we have $MST=⋃pch_{i}$. If the $MST_{i}$ is the MST of all nodes $s_{i}s\in pch_{i}$, then $MST_{i}=pch_{i}$ due to the characteristics of a convex hull. It is obvious that $L_{MST}\geq \sum L_{MST_{i}}$. Let $ss_{i}$ denote the straight skeleton built within $pch_{i}$. Theorem 3 ensures $L_{ss_{i}}<L_{MST_{i}}$, which implies $\sum L_{ss_{i}}<\sum L_{MST_{i}}\leq L_{MST}$ and $N_{OASS}<N_{MST}$. According to Theorem 4, $A_{MST}$ is a 3-opt; therefore, the approximation ratio of OASS is 3.

**Case 2**: the topology of graph *G* can’t be decomposed to a series of convex hulls.

In this case, there exists at least a subtree t∈MST, which is not a pch, such that $MST=⋃pch_{i}⋃t$. Similar to the previous case, we can get $\sum L_{ss_{i}}+L_{t}<\sum L_{MST_{i}}+L_{t}\leq L_{MST}$ and $N_{OASS}<N_{MST}$. According to Theorem 4, OASS is a 3-opt approximation algorithm.  ☐

We call an approach $A_{t}$, if $A_{t}$ deploys RNs along a tree *t* for connectivity restoration.

**Theorem** **6.** If a tree t has property 1, then $N_{A_{t}}<\frac{\sqrt{3}}{2}N_{A_{MST}}$.

**Proof.**  According to literature [27] and Theorem 3, if a shortest tree *t* has property 1, then we have $\frac{\sqrt{3}}{2}L_{MST}\leq L_{t}\leq L_{MST}$.

According to Equations (5) and (6), if $\frac{\sqrt{3}}{2}L_{MST}=L_{t}$, then we have

(9) $$\begin{array}{cc} N_{A_{MST}}\times \frac{\sqrt{3}}{2} & =\left(L_{MST}-2n+2\right)\times \frac{\sqrt{3}}{2}\\ & =\frac{\sqrt{3}}{2}L_{MST}-\sqrt{3}n+\sqrt{3}\\ & >\frac{\sqrt{3}}{2}L_{MST}-3n+4=N_{A_{t}}. \end{array}$$

Therefore, this theorem holds.  ☐

It is apparent that a straight skeleton has property 1 as described in Section 3. More importantly, we give the comparison between the length of the SMT and that of a straight skeleton in Table 3. It is obvious that each straight skeleton is almost as long as an SMT in every scenario that implies the fact that a straight skeleton is closely approximating the SMT. In addition, if the topology of the disconnected network can be decomposed into a series of convex hulls, then, according to Equations (2), (4) and Theorem 6, one can deduce that the approximation ratio of OASS can be reduced to $\frac{3\sqrt{3}}{2}$ with the value of *r* properly chosen.

It is worth mentioning that OASS can offer the optimum solution even in the special case that all representatives line up. In this case, OASS will deploy RNs between each pair of representatives sequentially. Although Theorem 5 ensures that, at most three times, the minimum number of RNs are required to restore the connectivity; in fact, OASS can achieve the optimum RNs deployment.

**Theorem** **7.** The complexity of OASS is O(nlogn).

**Proof.**  The OASS consists of three-phase processes, which are Ad-Prim, OA and SSIC, respectively. We give individual complexity analysis to prove that the complexity of OASS is O(nlogn).

In phase one, since Ad-Prim is an adapted construction algorithm for the MST with one rule that circles formed by nodes on the same $C_{i}$ should be ignored, and the complexity for Ad-Prim should be no more than that of Prim, which is O(nlogn) [25].

In phase two, OA discovers all obstacle–avoid paths in O(ε) while there are obstacles on MST, where ε is an constant.

In phase three, SSIC employs straight skeletons within pchs as relay node deployment routes. Suppose the number of $pch_{i}s\in MST$ is *k* such that $pch_{i}⋂pch_{j}=s_{l}$, where j=i+1. Therefore, we have $\sum |pch_{i}|=n+k-1$ that implies there are $\frac{n+k-1}{k}$ nodes for each pch on average. The construction of a convex hull, which is a pch in our case, costs no more than $O(\frac{n+k-1}{k}\log\frac{n+k-1}{k})$ [29]. Thus, *k* convex hulls will cost no more than $O\left(k\times \frac{n+k-1}{k}\log\frac{n+k-1}{k}\right)=O\left(n\log n\right)$, where 2≤k<n. In addition, each straight skeleton is built with a cost no more than O(nlogn) [28]. Since each straight skeletons can be built when a $pch_{i}$ is established, all straight skeletons will be established with the complexity of O(nlogn).

To sum up, each phase of OASS costs no more than O(nlogn); therefore, the complexity of OASS is O(nlogn).  ☐

### 5.2. Validation Experiment

We first give the comparison between the SMT and the straight skeleton in terms of the Euclidean distance. Then, performance metrics and baseline approaches are introduced. At last, simulation results and comparison of the generated topology quality are presented.

#### 5.2.1. Comparison between the SMT and the Straight Skeleton

Our tests were built in Euclidean space on some of Soukup examples [26]. In Table 3, we compare straight skeletons with Steiner Minimal Trees (SMTs). As illustrated in Table 3, the length of a straight skeleton is infinitely approximating to that of an SMT in all cases.

#### 5.2.2. Experiment Setup, Performance Metrics and Baseline Approaches

A simulation environment is implemented and validated in C++ environment on NS2.34. The experiments are conducted in 2000 m × 2000 m square area. Over 200 sensor nodes are deployed in this area. Overall, 30 random topologies in the given area are taken. For each topology, obstacles are randomly scattered over the deployment area. It is observed that, with 95% confidence level, the results are within 5–10% of the sample mean. The parameters and baseline approaches are introduced to evaluate the performance of the proposed solution are listed as follows.

**Communication range of relays (r)**: The performance of OASS is affected by *r*, basically longer distances between components require more RNs.

**Number of components $\left(N_{c}\right)$**: Intuitively, a large number of components require a larger RN count.

**Number of RNs $\left(N_{rn}\right)$**: A better approximation algorithm for the optimization of RN placement require less RNs than other strategies.

We compare the performance of OASS with the following four baseline approaches. The first one is ORNP [9] that employs the center of mass and 3-star to establish a relay node deployment route. The second one is RLC-GBP [14],which is short for Restore Relay Lost Connectivity using zero Gradient Based Point solution, deploys RNs toward a certain point of the deployment area. The third one is a straight skeleton based approach, named GSR [12]. IO-DT [11], the fourth one, is based on Delaunay triangulation.

**ORNP**: It is a two-phase algorithm. In phase one, 3-stars are constructed as many as possible. In phase two, the center of mass (CoM) of all remaining components is calculated. Then, each 3-star deploys RNs toward the CoM such that the connectivity is restored.

**RRLC-GBP**: It is one of $A_{tc}$. Similar to OASS, RRLC-GBP uses an MST based algorithm to find all representatives. Then, a distance function F with respect to the coordinates of each representative is constructed in order to find a specific point zero gradient point p such that the total distance from all representatives to p is minimized. At last, each representative deploys RNs toward the point p.

**GSR**: It is a straight skeleton based algorithm. According to the convex hull of all representatives, only one straight skeleton is constructed. Then, based on the position of each representative, RNs are populated along the straight skeleton until the connectivity is restored.

**IO-DT**: It is a three-phase algorithm. In phase one, the MST is built among all disjointed components. In phase two, along the MST, the Delaunay triangulation is employed based on which the Fermat weight of each triangle is calculated. In the last phase, according to the Fermat weight of each triangle the corresponding edges of which are used for inter-triangle connections or inner-triangle connections. Once the tree *t* that consists of triangles is built, RNs will deployed along *t*.

It is worth mentioning that ORNP, GSR and IO-DT don’t find the optimal solution to the representative selection problem. According to Theorem 1, the tree *t* built by OASS is shorter than that of ORNP, GSR and IO-DT. Furthermore, ORNP, RRLC-GBP, GSR and IO-DT assume that the deployment area is flat without obstacles, which is not realistic. In summary, OASS is developed in real scenarios with the presence of obstacles, the RNs required by which is less than ORNP, RRLC-GBP, GSR and IO-DT due to the tree built by OASS is shorter and the proper selection on representatives as well. Furthermore, compared with baseline approaches, OASS populates RNs around the obstacles in the shortest way. In order to show the advantage in the number of RNs consumed of OASS, we use OA to help all baseline approaches to avoid obstacles in our simulations.

#### 5.2.3. Simulation Results and Comparison of the Generated Topology Quality

Several configurations with different combinations of $N_{c}$ and *r* are simulated. We change the value of $N_{c}$ from 4 to 8, while *r* varies from 50 to 190 with increment of 20. The results of individual experiments for 30 topologies are then averaged.

**Figure 6:**
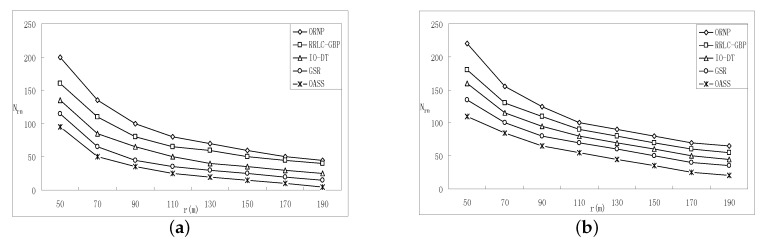
The effect of varying *r* on the performance of OASS compared with all baseline approaches w.r.t. (**a**) when $N_{c}=4$; (**b**) when $N_{c}=6$.

**Figure 7:**
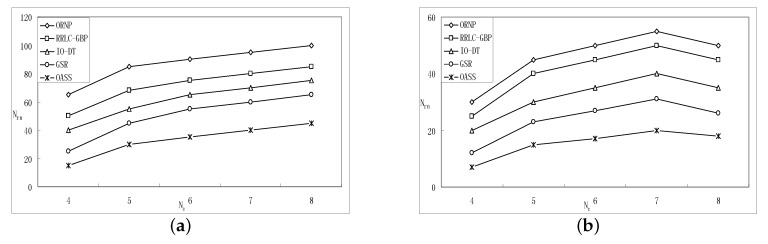
The effect of varying $N_{c}$ on the performance of OASS compared with all baseline approaches w.r.t. (**a**) when r=50 m; (**b**) when r=190 m.

Figure 6 and Figure 7 give the performance comparison between OASS and all baseline approaches, and OA is employed by all approaches to avoid obstacles.

Figure 6a,b show the required number of RNs while varying node communication range with $N_{c}=4$ and $N_{c}=6$, respectively. It is obvious that, as the communication range increases, RNs required for each approach decrease. It is clear that RRLC-GBP consumes less RNs than ORNP due to the fact that, compared with CoM, the zero gradient point is the point such that sum distance from all representatives to which is minimized. This is also confirmed in the simulation results, as shown in Figure 6a,b. Furthermore, the GSR shows better performance than ORNP, RRLC-GBP and IO-DT. The reason for that is: (1) ORNP and RRLC-GBP, as $A_{tc}$s, will repair the connectivity with a tree longer than the straight skeleton built by GSR as proved earlier; (2) the structure established by IO-DT that consists of 3-stars is similar to a straight skeleton; however, the RNs required are more than that of a straight skeleton. The OASS shows an excellent result compared with ORNP, RRLC-GBP, GSR and IO-DT as the communication range intensively increases. The reason is that a small count of RNs is deployed from all representatives along the tree that consists of straight skeletons along with the proper representative selection, as proved earlier.

Figure 7a,b show the required number of RNs while varying the number of representatives range with r=100 and r=200, respectively. It is obvious that, as the number of representative increases, the number of RNs required for each approach grows when r=100 as shown in Figure 7a. However, when *r* reach 200 m and $N_{c}=7$, the RN count drops for all approaches as the $N_{c}$ grows as shown in Figure 7b. The reason for that is the dense population of components shortens the inter-component distances, which implies that the RNs required are less. Again, Figure 7a,b confirm the advantage of OASS over ORNP, RRLC-GBP, GSR and IO-DT in RN count as the number of components increases.

It is obvious that, in the best case (e.g., the topology consists of convex hulls, all straight skeletons are not intersecting with obstacles), the OASS requires the minimum RN count. However, in the worst case (i.e., the topology not only consists of convex hulls, some straight skeletons are inevitable intersecting with obstacles), Theorem 5 guarantees that the RN count required by OASS is only three times that of the optimum.

We then give a comparison to show the topological advantages of OASS over RRLC-GBP in terms of distance (i.e., hops between every pair of representatives), delivery latency and balanced traffic load of the repaired network.

**Figure 8:**
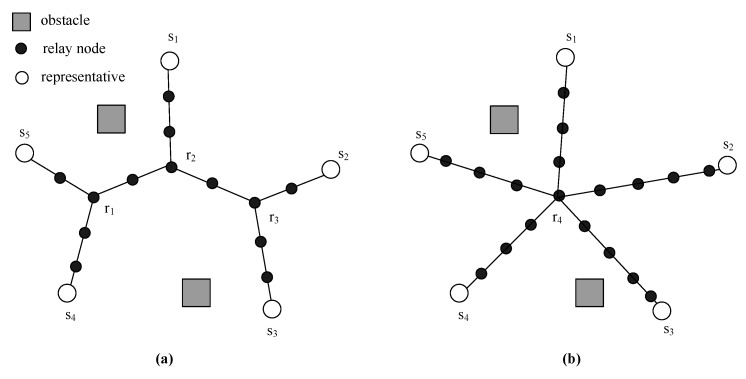
The comparison of the resulting topologies obtained by (**a**) OASS and (**b**) Restore Relay Lost Connectivity using zero Gradient Based Point solution (RRLC-GBP).

**Distance (hops)**: Figure 8 shows the comparison on the resulting topologies obtained by OASS and RRLC-GBP with the same setup. Accordingly, Table 4 presents the number of hops between every two segments in both topologies. We assume bidirectional data delivery between two representatives use the same path, where *A* denotes OASS as *B* denotes RRLC-GBP. Obviously, OASS deploys less RNs to forward data than RRLC-GBP. Overall, OASS requires 152 hops for delivery between all pairs of representatives, which is 22 hops less than that of RRLC-GBP. Therefore, OASS results in a shorter topology, which implies that data delivery requires less overall latency than that of RRLC-GBP.

**Delivery latency**: Since the delivery latency mostly results from data delivery via multi-hop links, the more hops a link processes, the more delivery latency will be generated. According to Table 4, it is obvious that the delivery latency of OASS is much less than that of RRLC-GBP.

**Balanced Traffic Load**: As shown in Figure 8a, it is clear that each pair of representatives should communicate with each other via relay nodes $r_{1}$, $r_{2}$ and $r_{3}$, which serve as rendezvous points of each communication path, while relay node $r_{4}$ is responsible for all communications between each pair of representatives as shown in Figure 8b. Statistically, about 32% of total traffic load are transferred via relay nodes $r_{1}$ and $r_{3}$, respectively, while relay node $r_{2}$ transfers 36% of which. However, 100% traffic load are transferred via relay node $r_{4}$. It is no doubt that $r_{1}$, $r_{2}$ and $r_{3}$ scatter the traffic load of $r_{4}$. In addition, the advantage of OASS over RRLC-GBP is quite obvious because the relay node as $r_{4}$ will die much earlier than other newly deployed ones that require another time of relay node deployment at the position where $r_{4}$ is located sooner or later.

## 6. Conclusions

The connectivity has significance in WSNs. Once the connectivity is lost, relay nodes are deployed to build an SMT such that the inter-component connection is reestablished. Most of the previous works assumed that the deployment area is flat without obstacles. However, it is not realistic. A lot of existing strategies chose the representative of each component in a random way or in the shortest-distance based manner. Both ways of representative selection could potentially increase the length of the SMT such that more RNs are required. In this paper, we propose a novel connectivity restoration strategy OASS, which employs both the polygon based representative selection with the presence of obstacles and the straight skeleton based SMT establishment. The OASS is proved to be a 3-opt approximation algorithm with the complexity of O(nlogn), and the approximation ratio can reduce to $\frac{3\sqrt{3}}{2}$ while it satisfies a certain condition. The theoretical analysis and simulations show that the performance of the OASS is better than other strategies in terms of the relay count and the quality of the established topology (i.e., distances between components, delivery latency and balanced traffic load) as well.

Although the OASS is a highly efficient algorithm, it is designed only for connectivity restoration problems in two-dimensional scenarios. In addition, if the deployment area is full of obstacles, then the OASS may not place the least number of relay nodes to restore the connectivity. In the future, we should address the three-dimensional connectivity restoration problem with the presence of massive obstacles.

## Figures and Tables

**Table 1:** Comparison of some contemporary heuristic algorithms for connectivity restoration in Wireless Sensor Networks (WSNs) through Relay Nodes (RNs) placement.

Authors	Approximation Ratio	Complexity
Misra et al. [8]	12.4	$O(n^{2k-2}+n^{2k+1}\log n)$
Lloyd et al. [4]	7	O(nlogn)
Yang et al. [7]	6.43	$O(n^{2k-2}+n^{2k+1}\log n)$
Misra et al. [6]	6.2	$O(n^{2k-2}+n^{2k+1}\log n)$
Tang et al. [3]	4.5	Not available
Efrat et al. [10]	3.11	$O(n^{2k-2}+n^{2k+1}\log n)$
Cheng et al. [5]	3	$O(n^{4})$
Chen et al. [2]	3	$O(n^{3})$
Wang et al. [9]	3	$O(n^{3})$
OASS (this paper)	3	O(nlogn)
OASS satisfying a certain condition (this paper)	$\frac{3\sqrt{3}}{2}$	O(nlogn)
Cheng et al. [5]	2.5	$O(n^{3}q_{p}^{2})$

**Table 2:** Notions.

Symbols	Descriptions
$P_{C_{i}}$	the polygon consists of nodes on the perimeter of $C_{i}$
$CH_{O}$	the convex hull of an obstacle O
$P_{s_{i},s_{j}}$	a path from $s_{i}$ to $s_{j}$
$P_{s_{1}s_{2}}^{O}$	a shortest path from $s_{i}$ to $s_{j}$ around the obstacle O
$d_{s_{i}s_{j}}$	the Euclidean distance of an edge $s_{i}s_{j}$
$L_{G}$	the length of the graph *G* in Euclidean space, that is $L_{G}=\sum_{e\in G}d_{e}$
$N_{A}$	RNs consumption function of the approach *A*

**Table 3:** Steiner Minimal Tree (SMT) vs. Straight Skeleton.

Number of Soukup Example	Number of Nodes	SMT	Straight Skeleton
EX.1	5	165.43	166.57
EX.2	6	155.06	156.04
EX.3	6	158.60	159.05
EX.4	6	130.15	131.03
EX.5	9	163.20	163.85
EX.6	9	128.50	129.10
EX.7	12	221.20	222.06
EX.8	14	122.02	122.64
EX.9	3	115.54	116.32
EX.10	10	164.26	165.20
EX.11	62	382.56	383.62
EX.12	14	172.30	173.23
EX.13	3	104.15	105.10
EX.14	5	182.92	183.05
EX.15	4	50.30	51.22

**Table 4:** OASS vs. Restore Relay Lost Connectivity using zero Gradient Based Point solution (RRLC-GBP) in distance (hops).

Hops	$s_{1}$		$s_{2}$		$s_{3}$		$s_{4}$		$s_{5}$	
	A	B	A	B	A	B	A	B	A	B
$s_{1}$	0	0	7	9	8	9	8	8	7	8
$s_{2}$	7	9	0	0	5	10	9	9	8	9
$s_{3}$	8	9	5	10	0	0	10	9	9	9
$s_{4}$	8	8	9	9	10	9	0	0	5	7
$s_{5}$	7	8	8	9	9	9	5	7	0	0
Sum	30	34	29	37	32	37	32	33	29	33
Ave.	6	6.8	5.8	7.4	6.4	7.4	6.4	6.6	5.8	6.6
